# Histopathological examination of congenital corneal staphyloma and prognosis after penetrating keratoplasty

**DOI:** 10.1097/MD.0000000000021892

**Published:** 2020-10-02

**Authors:** Yu Wan, Gege Xiao, Ting Yu, Pei Zhang, Jing Hong

**Affiliations:** Department of Ophthalmology; Beijing Key Laboratory of Restoration of Damaged Ocular Nerve, Peking University Third Hospital, Beijing, China.

**Keywords:** anterior segment dysgenesis, congenital corneal staphyloma, histopathological manifestations, penetrating keratoplasty, ultralarge button graft

## Abstract

The aim of this study was to investigate the histopathological manifestations of congenital corneal staphyloma accompanied by anterior segment dysgenesis and evaluate the prognosis after penetrating keratoplasty with an ultralarge button graft.

We retrospectively studied 8 pediatric patients with large congenital corneal staphylomas in the Department of Ophthalmology of Peking University Third Hospital, China, between September 2014 and December 2018. All patients underwent penetrating keratoplasty with ultralarge button grafts, as well as additional operations according to the abnormality of each eye. Pathological investigations of all samples obtained during penetrating keratoplasty were performed with hematoxylin and eosin staining.

The main clinical characteristic of congenital corneal staphyloma was an extremely opaque and ectatic cornea. Histopathological examination showed abnormal corneal epithelia and stroma and an absence of Bowman membrane, Descemet membrane, and the endothelium. Different severities of anterior segment dysgenesis, presenting as various histopathological manifestations, were observed in all cases. Several postoperative complications occurred after penetrating keratoplasty in some of the patients; however, the complications were discovered and treated accordingly in a timely manner. Six patients achieved good visual outcomes and a satisfactory cosmetic appearance after penetrating keratoplasty. One patient eventually lost the transparency of the button because of corneal neovascularization, and 1 patient lost visual function because of retinal detachment.

Congenital corneal staphyloma combined with anterior segment dysgenesis can exhibit various manifestations on histopathological examination. Penetrating keratoplasty with an ultralarge button graft seems to be a suitable treatment for congenital corneal staphyloma to obtain good functional and aesthetic prognoses.

## 1 Introduction

The protrusion of an opaque cornea at birth is a very rare condition.^[1]^ Previous studies have suggested that the prevalence of congenital corneal opacities ranges from 3/100,000 to 6/100,000 newborns.^[2]^ Congenital corneal staphylomas may account for approximately 11% of congenital corneal opacities.^[3]^ Although the incidence is rare, congenital corneal staphyloma is a lifelong vision-threatening disease associated with a poor visual prognosis and a seriously altered facial appearance.[^4^,^5^]

The clinical features of congenital corneal staphyloma include severe corneal opacification and anterior corneal protrusion. The anterior segment of the involved eye is usually anomalous.^[6]^ Typical histopathological manifestations of congenital corneal staphyloma include the absences of Bowman membrane, Descemet membrane, and the endothelium. The iris is usually atrophic and adherent to the posterior surface of the cornea.^[1]^ However, some congenital corneal staphylomas present with anterior segment dysgenesis, which exhibits various histopathological manifestations. This combination of disorders increases the difficulty of establishing an accurate diagnosis and early treatment regimen. Due to the extremely rare incidence of large congenital corneal staphylomas combined with anterior segment dysgenesis, very few studies have illustrated the various histopathological manifestations in detail.

Currently, there lacks a universally recognized treatment for congenital corneal staphyloma. The fates of most eyes are evisceration or enucleation.[^1^,^5^,^7^,^8^,^9^] However, the psychological trauma and the physical disability caused by evisceration or enucleation are quite extreme.[^10^,^11^,^12^] Penetrating keratoplasty for mild forms of congenital corneal staphyloma has been reported to improve the functional and esthetic prognosis,[^13^,^14^] but in severe cases, the incidence of graft failure is high.[^15^,^16^] Thus, successful therapeutic practices and proper surgical design are of great importance to determine an optimal treatment and enhance prognosis.

In this study, we present the various histopathological manifestations of large congenital corneal staphylomas combined with anterior segment dysgenesis. We further evaluate the prognoses after penetrating keratoplasty with ultralarge button grafts.

## 2 Materials and methods

### 2.1 Patients

We retrospectively studied 8 pediatric patients with congenital corneal staphylomas at Peking University Third Hospital, Peking University Eye Center, between September 2014 and December 2018. The cohort included 6 boys and 2 girls, aged 5 months to 1 year. This investigation was approved by the Institutional Review Board of Peking University Third Hospital, and informed consent was obtained from the representatives of all patients.

### 2.2 Examination and surgery

All patients underwent basic ocular examinations including visual acuity (if possible), intraocular pressure (IOP), exophthalmos, eye position and eye movement evaluations. The structure of the anterior segment was observed with a slit-lamp examination and, when possible, ultrasound biomicroscopy (UBM) and anterior segment optical coherence tomography (AS-OCT). The posterior segments were evaluated by a B-type ultrasound scan. Systemic examination and biochemistry tests were conducted to identify other systemic diseases. All of the patients underwent penetrating keratoplasty under general anesthesia. Additional operations, such as iridectomy, lens extraction, anterior vitrectomy, goniosynechialysis, and gonioplasty, were also conducted according to the abnormality of each eye.

### 2.3 Histopathological examination

The excised corneal tissues were sent for histopathological examination. The sections were stained with hematoxylin and eosin (HE), and then examined by light microscopy.

### 2.4 Postoperative follow-up

All 8 patients received close observation and attentive nursing care postoperatively. Antibiotics, corticosteroid, immunosuppressant, and artificial tears were topically used after surgery. No systemic immunosuppressive therapy was applied. In addition, specific therapies were conducted according to different postoperative complications. The follow-up period ranged from 4 months to 4 years. The follow-up frequency ranged from 1 week to 6 months. Prognostic evaluations included visual acuity, IOP, corneal state, and intraocular condition.

## 3 Results

Eight eyes with congenital corneal staphyloma were retrospectively studied (Table 1). The mean age of the patients was 7 months and ranged from 5 months to 1 year. In all patients, the protruding opaque mass on the eye was present at birth. Most of the patients presented no systemic disease or other congenital defects, except for 1 patient who had polydactyly of the right hand as well as a urethral cleft. All of the patients were treated by penetrating keratoplasty with ultralarge button grafts. The follow-up period ranged from 4 months to 4 years (mean, 1.7 years).

**Table 1:**
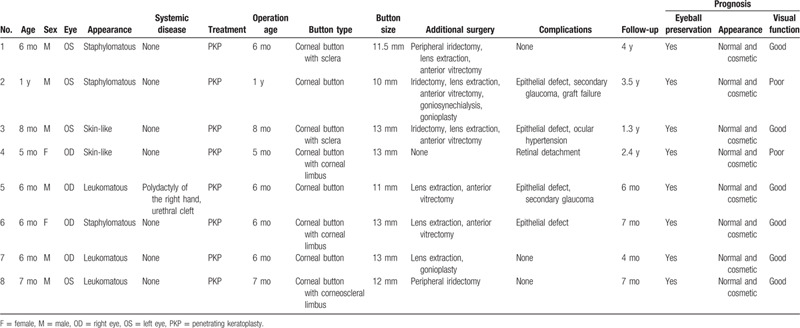
Clinical Characteristics, Surgical Interventions, and Postoperative Prognoses of the 8 Patients.

### 3.1 Clinical observation

**Figure 1:**
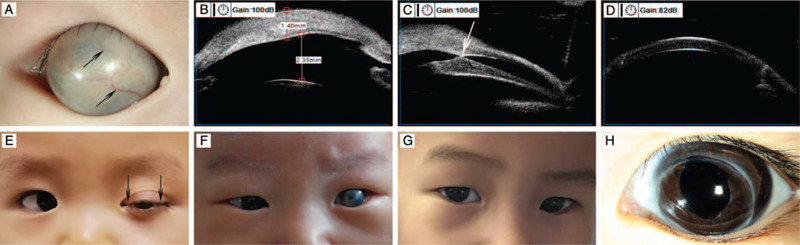
Clinical observations of the eye with congenital corneal staphyloma before and after penetrating keratoplasty. (A) Preoperative anterior segment photograph of congenital corneal staphyloma showed a large skin-like mass protruding between the eyelid. Growth of blood vessels could be seen on its surface (black arrows). (B, C) Preoperative ultrasound biomicroscopy (UBM) showed an opaque and thickened cornea. The iris was adhered to the posterior surface of cornea (white arrow). There is no significant reduction of the anterior chamber depth. (D) Postoperative UBM showed the corneal status after penetrating keratoplasty. (E) Postoperative photograph of a child with persistent epithelial defects. Temporary eyelid sutures were conducted at the inner and outer canthus (black arrows) to reduce the exposure area; meanwhile, the sight could still pass through the center part of the graft. (F) Preoperative photograph of patient 1 with congenital corneal staphyloma, exhibiting an abnormal appearance. (G) Photograph of patient 1 taken 4 years after the operation, showing a satisfactory cosmetic appearance postoperatively. (H) Postoperative anterior segment photograph showed that the graft remained clear 4 years after the operation.

On ocular examination, all the patients exhibited a protruding opaque mass on the cornea (Fig. 1). Two were skin-like, 3 were staphylomatous, and the other 3 were leukomatous (Table 1). The UBM and AS-OCT showed an opaque and thickened cornea. The iris was adhered to the posterior surface of cornea (Fig. 1). The anterior chamber depth was reduced or normal, varying by the severity of each dysgenesis. The B-mode ultrasound scan showed normal posterior segments.

### 3.2 Penetrating keratoplasty and intraoperative conditions

All patients underwent penetrating keratoplasty with an ultralarge donor corneal/corneoscleral button. To remove the lesions thoroughly, all masses were excised together with 1 mm peripheral normal cornea. After excising the corneal mass, the abnormal anterior segments with varying severities were exposed, and additional operations were conducted depending on the abnormality in each eye (Table 1). Iridectomy was conducted in case the iris was abnormal and tightly adhered to the posterior surface of the cornea. If there were some remnants of healthy iris, the normal iris was carefully separated and preserved as much as possible. Anterior vitrectomy was conducted in patients with severe anterior segment dysgenesis, to avoid postoperative complications such as vitreous hernia and intraocular hypertension. The lens was extracted if it was opaque, degenerated, or tightly attached to the posterior surface of the cornea. Goniosynechialysis and gonioplasty were also conducted in some cases when necessary, according to each specific situation.

### 3.3 Histopathological examination

**Figure 2:**
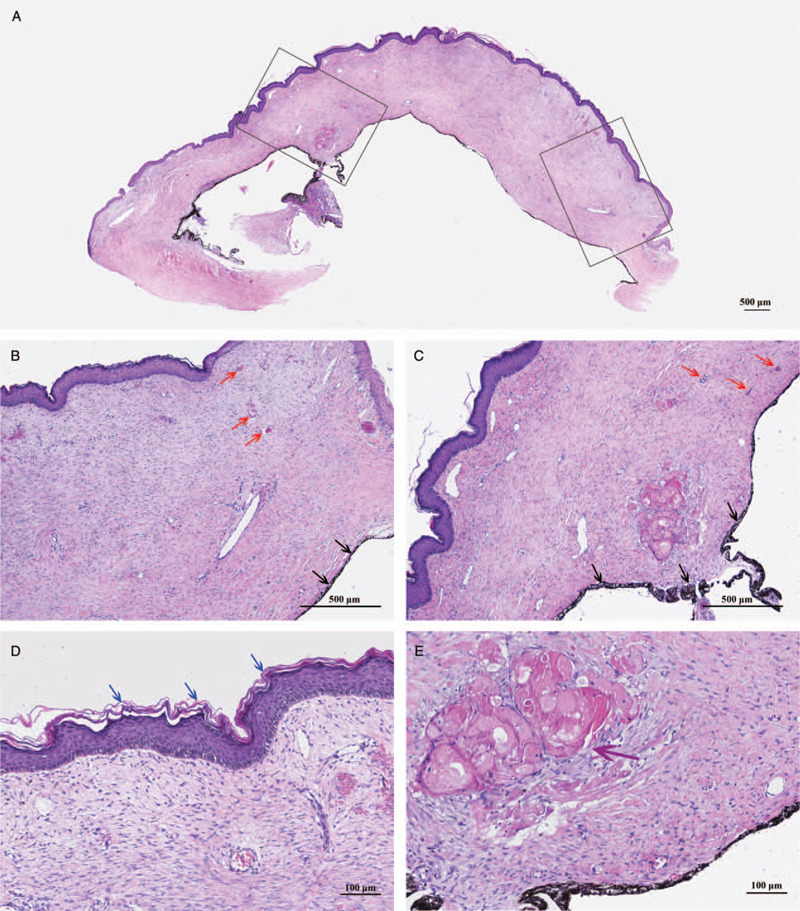
Histopathological manifestations of congenital corneal staphyloma (HE staining). (A) The whole cornea scanning image of the staphyloma. The corneal tissue was thickened and had a rough surface. (B, C) The corneal stroma was thickened and presented connective tissue-like changes. The dysplastic iris adhered to the posterior surface of the cornea (black arrows). Irregular collagen fibrils, fibroblast-like cells, and various blood vessels (red arrows) were observed. (D) The corneal epithelium was hyperplastic, with squamous metaplasia (blue arrows). Bowman membrane was absent. (E) The dysplastic lens tissue was embedded in the cornea (purple arrow). Descemet membrane and the endothelium were absent.

Postoperatively, the excised tissue was sent for histopathological examination (Fig. 2). Histopathology with HE staining revealed abnormal corneal epithelia and stroma. Bowman membrane, Descemet membrane, and the endothelium were absent. Anterior segment dysgenesis was observed in all patients, but the histopathological manifestations varied (Table 2).

**Table 2:**
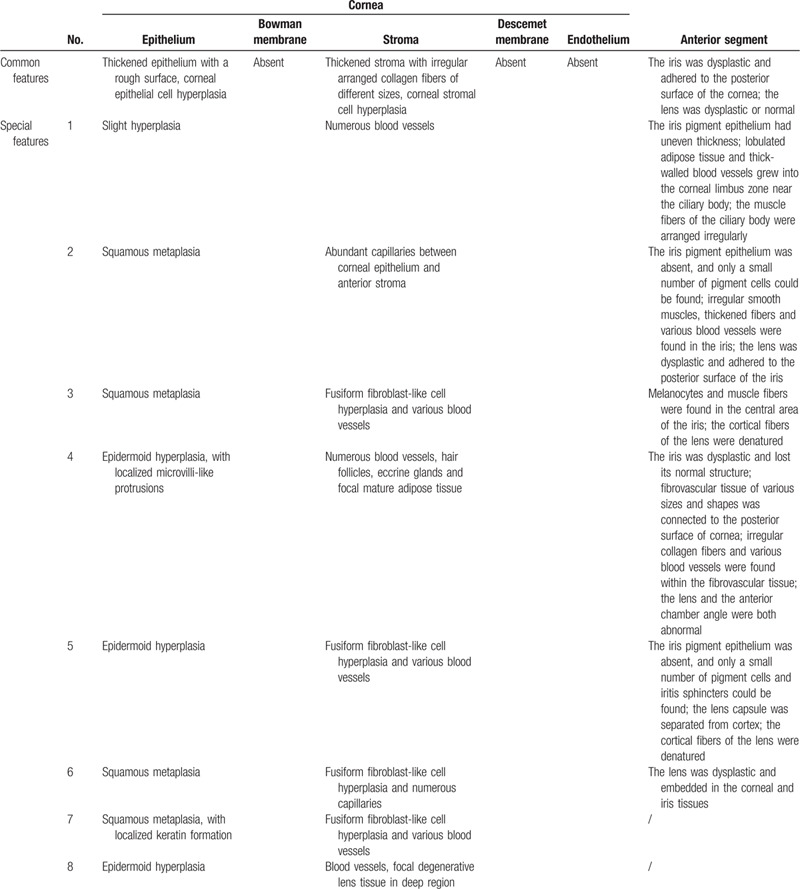
Histopathological Manifestations of Congenital Corneal Staphylomas in the 8 Eyes.

### 3.4 Postoperative complications and prognosis

Three patients recovered well after surgery and showed no postoperative complications. Postoperative complications occurred in 5 patients but were discovered and treated in a timely manner (Table 1). Epithelial defects occurred in 4 eyes. Two were identified half a month after surgery, one was identified half a year after surgery, and the other was identified 1 year after surgery. For children with persistent epithelial defects, temporary eyelid sutures for an average period of 3 months were conducted, and achieved a good effect in corneal epithelial defects healing. Eyelid sutures were only conducted at the inner and outer canthus to reduce the exposure area; meanwhile, the sight could still pass through the center part of the graft (Fig. 1). The IOP of each patient was closely monitored after the surgery. Ocular hypertension occurred in 3 eyes. One soon returned to normal with timely medical management and remained controlled. The other 2 eyes developed secondary glaucoma, and glaucoma valve implantation was performed. The IOP returned to normal after the operation and the hypertension remained controlled. Six of the 8 patients achieved a satisfactory cosmetic appearance and good visual outcomes (Fig. 1). One patient lost sight in her operated eye 6 months postoperatively because of retinal detachment. One patient lost transparency of the button 2 years after surgery due to corneal neovascularization.

## 4 Discussion

Congenital anterior staphyloma is a developmental abnormality characterized by an opaque and protruding cornea and usually results in a poor prognosis with low visual function.[^4^,^17^] Because of its rarity, there are few studies on congenital anterior staphylomas, and the understanding of this disease is still limited. In the present study, we retrospectively studied 8 cases of congenital corneal staphyloma, described the histopathological features, and analyzed the prognosis after penetrating keratoplasty with an ultralarge button graft.

The typical clinical feature of congenital corneal staphyloma is a severely opaque and protruding cornea.^[8]^ In our study, the 8 eyes all fit the clinical features of congenital corneal staphyloma. Three were typically staphyloma-like, and the others had an atypical leukomatous or skin-like appearance. Microscopically, the histopathological characteristics of congenital corneal staphylomas include corneal ectasia, with remnants of an atrophic iris adhered to its posterior surface. The epithelium is intact but may show keratinization secondary to exposure.^[13]^ The corneal stroma is disorganized, vascularized, and hypercellular.^[18]^ Bowman membrane, Descemet membrane, and the endothelium are usually absent.^[15]^ Similar corneal histopathological features were found in our cases, but the manifestations of anterior segment dysgenesis varied. The severity of iris dysplasia, the degree of lens involvement, the anterior chamber depth, and the existence of other ocular abnormalities varied among patients. The pathogenesis of congenital corneal staphyloma may partly explain this phenomenon. Congenital corneal staphyloma is presumed to be caused by the failure of the normal migration of neural crest cells.^[18]^ During the fifth to sixth week of gestation, separation of the lens vesicle and basement membrane of the surface ectoderm occurs, followed by 3 successive developmental stages. The first stage gives rise to the corneal endothelium and trabecular meshwork, the second stage gives rise to the corneal keratinocytes and corneal stroma, and the third stage produces the iris.^[3]^ The arrest of any of these developmental stages will induce anterior segment dysgenesis, and arrest of different developmental stages will lead to various manifestations.

In this study, all 8 patients accepted penetrating keratoplasty with ultralarge button grafts. Six patients achieved a satisfactory cosmetic appearance and good visual outcomes. Here, we recommend penetrating keratoplasty as an early treatment for congenital corneal staphyloma. Delayed intervention may result in dramatic growth of the abnormal tissue, abnormal ocular development, or even spontaneous phthisis bulbi.^[5]^ In addition, the corneal opacity may influence the normal development of vision and lead to amblyopia.^[19]^ As for the surgical procedures of penetrating keratoplasty, we thought there were several tips crucial for the postoperative prognosis of this disease. First, the lesions should be removed thoroughly. We resected the masses together with a little peripheral normal cornea to ensure the effect. Moreover, for the cases with severe anterior segment dysgenesis, thorough anterior vitrectomy is important to avoid postoperative complications such as vitreous hernia and intraocular hypertension. The abnormal iris should be excised; meanwhile, the healthy remnants should be preserved as much as possible. Complete separation of the anterior chamber angle is also important.

Several postoperative complications occurred in our patients, similar to previous reports.[^13^,^20^] One complication was ocular hypertension, which is considered to be a common clinical occurrence after penetrating keratoplasty.[^21^,^22^] Several factors were considered contributing factors to postoperative ocular hypertension in our cases, such as severe congenital anterior segment dysgenesis, additional surgeries combined with penetrating keratoplasty, surgical injuries to the anterior chamber angle when excising abnormal tissues and postoperative inflammation. Another postoperative complication was corneal epithelial defects, which was also frequently occur in the donor cornea after penetrating keratoplasty.^[23]^ Contributing factors for postoperative epithelial defects are considered to be ocular surface disorders, exposure secondary to eyelid abnormality, ocular cicatrizing disorders and limbal stem cell deficiency.^[24]^ Because most of our patients underwent penetrating keratoplasty with ultralarge button grafts to replace the diffusely diseased corneas, little or no host endothelial cells were preserved after surgery. The deficiency of limbal stem cells was considered the main contributing factor for postoperative epithelial defects in our cases. Additionally, one patient lost visual function because of retinal detachment three months postoperatively. This patient had the most severe degree of ocular dysgenesis among the 8 cases; no normal corneal tissue, corneal limbus tissue, iris tissue or lens tissue was found in this eye. Severe dysgenesis and complicated surgical procedures were considered contributing factors for her retinal detachment.[^25^,^26^] Moreover, atypical hyperplasia was found in this eye after surgery, and this might also have contributed to the eye dysfunction.

Due to special ocular features and a more active immune system, penetrating keratoplasty in children has a higher graft failure rate than penetrating keratoplasty in adults.[^27^,^28^,^29^] It has been reported that graft survival in children under 5 years old is <40% at 16 years postoperatively.^[30]^ In addition to age, congenital structural abnormalities of the anterior segments also markedly reduce the visual outcome after corneal transplantation.[^19^,^31^] Moreover, additional noncorneal surgical procedures, such as lensectomy and vitrectomy, at the time of penetrating keratoplasty are associated with graft failure in children.[^30^,^32^] In addition, the use of ultralarge button grafts for penetrating keratoplasty increased the risk for graft failure. It has been reported that a large donor cornea increases the risk of graft rejection since there is a higher number of dendritic Langerhans cells in the limbal region of the cornea than in the center. Furthermore, a large donor cornea is positioned relatively close to the limbal region and is therefore exposed to the antigen/antibody influences of the limbal vasculature.^[33]^

In brief, our 8 cases of congenital corneal staphyloma combined with anterior segment dysgenesis exhibited various histopathological manifestations. These histopathological findings may provide clues for the recognition of this disease and help to enhance the understanding of rare abnormalities. Although the risk of postoperative complications is high in young children and severe cases, satisfactory outcomes were obtained in most of our cases after penetrating keratoplasty with an ultralarge button graft. Therefore, early surgical treatment is still recommended as a priority option to obtain better development of the orbit and vision. For patients whose limbal stem cells are diffusely affected, the difficulty in corneal epithelial regeneration after transplantation is still a notable problem. Concurrent surgical procedures at the time of penetrating keratoplasty had a detrimental effect on graft survival, but they were necessary and ineluctable for patients who had multiple ocular abnormalities. A better understanding and management of these factors may improve the prognosis in the future.

## Author contributions

**Investigation:** Yu Wan.

**Methodology:** Yu Wan, Gege Xiao, Ting Yu, Pei Zhang, Jing Hong.

**Project administration:** Yu Wan.

**Resources:** Gege Xiao, Ting Yu, Jing Hong.

**Writing – original draft:** Yu Wan.

**Writing – review & editing:** Yu Wan, Gege Xiao, Jing Hong.

## References

[1] Salour H Owji N Sadeghipour A Congenital corneal staphyloma. J Ophthalmic Vision Res 2009;4:182–4. PMC3498567 23198071

[2] Ciralsky J Colby K Congenital corneal opacities: a review with a focus on genetics. Semin Ophthalmol 2007;22:241–6. 18097987 10.1080/08820530701745157

[3] Shigeyasu C Yamada M Mizuno Y Clinical features of anterior segment dysgenesis associated with congenital corneal opacities. Cornea 2012;31:293–8. 22157569 10.1097/ICO.0b013e31820cd2ab

[4] Bernuy A Contreras F Maumenee AE Bilateral, congenital, dermis-like choristomas overlying corneal staphylomas. Arch Ophthalmol 1981;99:1995–7. 7295148 10.1001/archopht.1981.03930020871011

[5] Kim MJ Choung HK Kim NJ Congenital corneal staphyloma treated by evisceration and primary implant placement: 3 cases. Can J Ophthalmol 2008;43:111–3. 18204493 10.3129/i07-183

[6] Zhang Y Zhou J Zhu D Ultrasonographic characteristics of congenital corneal staphyloma. J Med Ultrasonics (2001) 2016;43:291–3. 10.1007/s10396-015-0675-8 27033873

[7] Tanaka R Takenouchi T Uchida K Congenital corneal staphyloma as a complication of Kabuki syndrome. Am J Med Genet A 2012;158a:2000–2. 22786791 10.1002/ajmg.a.35453

[8] Schramm C Rohrbach JM Reinert S Amniotic bands as a cause of congenital anterior staphyloma. Graefes Arch Clin Exp Ophthalmol 2013;251:959–65. 23150045 10.1007/s00417-012-2197-z

[9] Matsubara A Ozeki H Matsunaga N Histopathological examination of two cases of anterior staphyloma associated with Peters’ anomaly and persistent hyperplastic primary vitreous. Br J Ophthalmol 2001;85:1421–5. 11734512 10.1136/bjo.85.12.1421 PMC1723813

[10] Soares IP Franca VP Evisceration and enucleation. Semin Ophthalmol 2010;25:94–7. 20590419 10.3109/08820538.2010.488575

[11] Fountain TR Goldberger S Murphree AL Orbital development after enucleation in early childhood. Ophthalm Plast Reconstr Surg 1999;15:32–6. 10.1097/00002341-199901000-00008 9949427

[12] Kord Valeshabad A Naseripour M Asghari R Enucleation and evisceration: indications, complications and clinicopathological correlations. Int J Ophthalmol 2014;7:677–80. 25161942 10.3980/j.issn.2222-3959.2014.04.17 PMC4137206

[13] Schanzlin DJ Robin JB Erickson G Histopathologic and ultrastructural analysis of congenital corneal staphyloma. Am J Ophthalmol 1983;95:506–14. 6837694 10.1016/0002-9394(83)90273-8

[14] Miller MM Butrus S Hidayat A Corneoscleral transplantation in congenital corneal staphyloma and Peters’ anomaly. Ophthalm Genet 2003;24:59–63. 10.1076/opge.24.1.59.13891 12660867

[15] Verschooten R Foets B De Ravel T Clinical spectrum of congenital corneal staphyloma: a case report. Bull Soc Belge Ophtalmol 2011;7–10. 22003758

[16] Jacob S Prakash G Ashok Kumar D Anterior segment transplantation with a novel biosynthetic graft. Eye Contact Lens 2010;36:130–6. 20093940 10.1097/ICL.0b013e3181cd1b14

[17] Al Judaibi R Abu-Amero KK Morales J Mutations of the CYP1B1 gene in congenital anterior staphylomas. Clin Ophthalmol 2014;8:445–8. 24591815 10.2147/OPTH.S53200 PMC3938497

[18] Leff SR Shields JA Augsburger JJ Congenital corneal staphyloma: clinical, radiological, and pathological correlation. Br J Ophthalmol 1986;70:427–30. 3718906 10.1136/bjo.70.6.427 PMC1041034

[19] Vanathi M Panda A Vengayil S Pediatric keratoplasty. Survey Ophthalmol 2009;54:245–71. 10.1016/j.survophthal.2008.12.011 19298903

[20] Karadag O Kugu S Erdogan G Incidence of and risk factors for increased intraocular pressure after penetrating keratoplasty. Cornea 2010;29:278–82. 20118781 10.1097/ICO.0b013e3181b6eb9e

[21] Mannis MJ Holland EJ Beck RW Clinical profile and early surgical complications in the Cornea Donor Study. Cornea 2006;25:164–70. 16371775 10.1097/01.ico.0000164832.69668.4b

[22] Orucoglu F Blumenthal EZ Frucht-Pery J Risk factors and incidence of ocular hypertension after penetrating keratoplasty. J Glaucoma 2014;23:599–605. 23429622 10.1097/IJG.0b013e31828700f5

[23] Ricci F Missiroli F Ciotti M Persistent epithelial defect after penetrating keratoplasty caused by adenoviral infectious keratitis. New Microbiol 2010;33:171–4. 20518280

[24] Mannis MJ Zadnik K Miller MR Preoperative risk factors for surface disease after penetrating keratoplasty. Cornea 1997;16:7–11. 8985626

[25] Park Y Kim MH Won JY Vitreoretinal complications after penetrating keratoplasty. Retina (Philadelphia, Pa) 2016;36:2110–5. 10.1097/IAE.0000000000001049 27115992

[26] Aiello LP Javitt JC Canner JK National outcomes of penetrating keratoplasty. Risks of endophthalmitis and retinal detachment. Arch Ophthalmol 1993;111:509–13. 8470985 10.1001/archopht.1993.01090040101041

[27] Al-Ghamdi A Al-Rajhi A Wagoner MD Primary pediatric keratoplasty: indications, graft survival, and visual outcome. J AAPOS 2007;11:41–7. 17307682 10.1016/j.jaapos.2006.09.012

[28] O’Hara MA Mannis MJ Pediatric penetrating keratoplasty. Int Ophthalmol Clin 2013;53:59–70. 10.1097/IIO.0b013e3182782a4b 23470589

[29] Low JR Anshu A Tan AC The outcomes of primary pediatric keratoplasty in Singapore. Am J Ophthalmol 2014;158:496–502. 24875001 10.1016/j.ajo.2014.05.020

[30] Lowe MT Keane MC Coster DJ The outcome of corneal transplantation in infants, children, and adolescents. Ophthalmology 2011;118:492–7. 20932584 10.1016/j.ophtha.2010.07.006

[31] McClellan K Lai T Grigg J Penetrating keratoplasty in children: visual and graft outcome. Br J Ophthalmol 2003;87:1212–4. 14507748 10.1136/bjo.87.10.1212 PMC1920761

[32] Karadag R Chan TC Azari AA Survival of primary penetrating keratoplasty in children. Am J Ophthalmol 2016;171:95–100. 27590122 10.1016/j.ajo.2016.08.031

[33] Yu AL Kaiser M Schaumberger M Perioperative and postoperative risk factors for corneal graft failure. Clin Ophthalmol 2014;8:1641–7. 25210433 10.2147/OPTH.S65412 PMC4155896

